# NT-proBNP change is useful for predicting weaning failure from invasive mechanical ventilation among postsurgical patients: a retrospective, observational cohort study

**DOI:** 10.1186/s12871-023-02039-7

**Published:** 2023-03-20

**Authors:** Yingying Zheng, Zujin Luo, Zhixin Cao

**Affiliations:** Department of Respiratory and Critical Care Medicine, Beijing Institute of Respiratory Medicine and Beijing Chao-Yang Hospital, Capital Medical University, Beijing, China

**Keywords:** Invasive mechanical ventilation, Weaning failure, Cardiovascular dysfunction, NT-proBNP, Postsurgical patients

## Abstract

**Background:**

To evaluate the predictive value of N-terminal prohormone B-type natriuretic peptide (NTproBNP) for weaning failure among patients undergoing major surgeries during spontaneous breathing trial (SBT), compared to traditional weaning parameters.

**Methods:**

The observational cohort study retrospectively included postsurgical patients who received IMV and underwent a 2 h SBT. According to weaning outcome, NTproBNP level at initiation (NTproBNP1) and at end of 2 h SBT(NTproBNP2), the ΔNTproBNP%, RSBI and MV were compared between weaning failure and weaning success group. Multiple logistical regression and ROC curve were used to evaluate the capability of NTproBNP to predict weaning failure.

**Results:**

Out of the 323 included postsurgical patients, 45 (13.9%) patients had failed weaning. The ΔNTproBNP% was a better predictor for weaning failure (AUC 0.744;95%CI,0.693–0.791) than NTproBNP1(AUC 0.639; 95%CI,0.580–0.694)), NTproBNP2(AUC 0.742, 95%CI,0.688–0.792) and other traditional weaning index such as RSBI (AUC 0.651; 95%CI, 0.597–0.703) and MV (AUC 0.552; 95%CI,0.496–0.607). The cutoff value of ΔNTproBNP% for predicting weaning failure was 23.3% with the sensitivity75.76% and specificity73.38%. The multiple logistic regression analysis found that ΔNTproBNP%>23.3% was an independent predictor of weaning failure.

**Conclusion:**

ΔNTproBNP% may be a useful marker for predict weaning failure for postsurgical patients, and it’s better to be more careful to withdraw from invasive mechanical ventilation for those postsurgical patients with ΔNTproBNP% >23.3%. The corresponding interventions to optimize cardiac function should be actively given to these patients.

## Introduction

Liberating critically ill patients from invasive mechanical ventilation (IMV) is a gradual and challenging process, any delay in ventilation removal may lead to ventilator acquired pneumonia and other possible side effects [1]. The spontaneous breathing test trial (SBT) is considered to be the most accurate method to predict the results of weaning, but the extubation failure rate is still high (15–20%) in patients who have passed SBT [2]. It has been reported that there were 14.5% of postoperative patients among the patients receiving mechanical ventilation in intensive care unit(ICU) [3]. Although postsurgical patients tended to have lower weaning failure rate compared to medical patients in our previous study [4], those patients still face much challenge during weaning process. There are multiple mechanisms of weaning failure [5–7]. Underlying cardiovascular dysfunction induced by the stress of weaning has been reported to play a key role [8–10]. In surgical patients, the etiologies leading to tracheal intubation and mechanical ventilation were different from the medical patients. In addition, surgeries, hemorrhage, and anaesthesia during surgery could have some adverse effect on the cardiac function [11, 12]. Intraoperative fluid infusion during surgery was also a challenge to cardiac function [13]. Underlying cardiac dysfunction during perioperative period is an prominent risk factor leading to the failure of weaning from invasive ventilator [14].

B-type natriuretic peptides are produced by cardiac ventricular myocytes in response to volume or pressure overload [15]. Two B-type natriuretic peptides are detectable in the circulation after proteolysis of prohormone B-type natriuretic peptide (proBNP): Brain natriuretic peptide(BNP) and N-terminal proBNP(NTproBNP) [16]. Plasma BNP level and NTproBNP have been considered as sensitive markers of cardiovascular dysfunction [17]. Recent data suggested that NTproBNP may predict the outcome of weaning from IMV for patients with respiratory illness [18], adult patients after cardiac surgery [19] or mix population [20–22]. However, there was considerable diversity in terms of populations evaluated, weaning and extubation methods, and outcomes analyzed, so the results of natriuretic peptides predicting weaning outcomes were divergent. And most the studies focused on the medical patients or mixed patients.

Nevertheless, the value of NTproBNP predicting the weaning outcome among patients with noncardiac surgery have not been studied. We therefore aimed to determine the value of NTproBNP as a predictor of weaning failure from IMV in noncardiac postsurgical patients. We hypothesized that NTproBNP would be a predictor of weaning failure in noncardiac postsurgical patients, compared to other traditional parameters related with weaning outcome, such as rapid shallow breathing index (RSBI), and minute volume (MV).

## Materials and methods

### Study design

This retrospective observational cohort study included postsurgical patients admitted to a 12-bed ICU of Beijing Chao-Yang Hospital in China between January 2013 to December 2019. The study was conducted in accordance with the principles of the Declaration of Helsinki, and the study protocol was approved by the ethics committee of the Beijing Chao-Yang Hospital, Capital Medical University (NO.2020-KE-94).

The need for informed consent was waived by the ethics committee of the Beijing Chao-Yang Hospital, Capital Medical University, because of the retrospective nature of the study.

### Inclusion criteria

All postsurgical patients intubated and mechanically ventilated for not less than 12 h were considered eligible for the study if they fulfilled the following [4, 23]: resolution of the underlying causes of acute respiratory failure; adequate cough reflex; absence of excessive tracheobronchial secretion; adequate oxygenation (e.g., arterial oxygen saturation > 90% or arterial oxygen tension/fraction of inspired oxygen [PaO_2_/FiO_2_] ≥ 150 mmHg, both on the FiO_2_ of ≤ 0.4 and the positive end-expiratory pressure of ≤ 8 cmH_2_O); adequate ventilatory status (e.g., respiratory rate [RR] ≤ 35 breaths/min with tidal volume ≥ 5 mL/kg of predicted body weight and no significant respiratory acidosis); stable hemodynamics (e.g., heart rate [HR] < 120 beats/min; systolic blood pressure [SBP], 90–160 mmHg; and no or minimal vasopressor use); adequate mentation (e.g., arousable or glasgow coma scale ≥ 13 with no continuous sedative infusions); body temperature < 38 ℃; hemoglobinemia ≥ 80 g/L; and acceptable electrolytes. The postsurgical patients included patients admitted to the ICU immediately after surgery and patients transferred to ICU within 1 week after surgery.

### Exclusion criteria

Age < 18 years; pregnancy; tracheotomy or other upper airway disorders; mechanically ventilated less than 12 h; abandoned before extubation; neuromuscular disease; decision to limit active treatment; chronic kidney disease; chronic heart failure; and incomplete data. The inclusion and exclusion criteria were described in our previous research [4, 23].

### Weaning protocol

A 2 h SBT was performed in all eligible postsurgical patients, which allowed the patients to breathe spontaneously through a T-tube circuit with the FiO_2_ set at the same level used during IMV while the patients were in a semi-recumbent position (45°). The SBT was the first trial for every patient. During the SBT, RR SBP, HR, pulse oxymetry, five-lead electrocardiographic tracing, and clinical signs were closely monitored. Arterial blood gases were analyzed at the beginning of the SBT.

A criteria for SBT failure were: (1)arterial pH < 7.32 with arterial carbon dioxide tension (PaCO_2_) ≥ 10 mmHg higher than baseline; (2)RR > 35 breaths/min or ≥ 50% higher than baseline; (3)peripheral oxygen saturation (SpO_2_) < 90% or PaO_2_ ≤ 60 mmHg at FiO_2_ ≥ 0.4; (4)HR > 140 beats/min or ≥ 20% higher/lower than baseline;(5) SBP > 180 or < 90 mmHg or ≥ 20% higher/lower than baseline; (6)use of accessory respiratory muscles, or thoracic-abdominal paradoxical movement; decreased consciousness, agitation, or diaphoresis. Patients free of these features at the end of SBT were considered to succeed the SBT and subsequently extubated.

Weaning failure was defined as SBT failure or reintubation within 48 h following extubation [24]. Weaning success was defined as extubation successfully and the absence of reintubation for more than 48 h following extubation. We share the same weaning protocol in our department, and this was described in our previous research [4, 23].

### Clinical outcome

The primary outcome was weaning failure. The secondary outcomes included length of stay in ICU, length of stay in hospital, and hospital mortality.

### Data collection

At enrollment, patients’ baseline characteristics were recorded: demographic data, acute physiology and chronic health evaluation II (APACHE II) score, IMV duration prior to SBT, medical history, surgery sites. In addition, vital signs, rapid shallow breathing index(RSBI), minute volume(MV), expired tidal volume(Vte), arterial blood gases and bedside echocardiography were recorded before SBT. After extubation, the following was recorded: success or failure of weaning, length of ICU stay, length of hospital stay, hospital mortality.

NTproBNP levels at the beginning and at the end of 2 h SBT were determined through immunofluorescence, with EDTA as the anticoagulant. Peripheral venous blood samples were drawn at initiation of the SBT to measure hemoglobin(Hb), albumin(ALB), creatinine, and β2 macroglobulin.

### Statistics

For continuous variables, Shapiro-Wilk tests were performed to determine the normality of the data distribution. Data were described as mean ± standard deviation (SD) and Student’s t test was used for normally distributed data. Data were expressed as median (25th-75th percentile) and the Mann-Whitney U-test was employed for non-normally distributed data. For comparing categorical data, described as frequencies and percentages, Chi square (χ^2^) test was performed. Receiver operator characteristic (ROC) analysis was used to determine the optimum cutoff value of studied markers for predicting weaning failure. Univariate and multiple logistic regression analysis were done to determine the risk factors for weaning failure. Multiple logistical regression analysis was performed with covariates which showed P ≤ 0.01 by univariate analysis, including ALB, Hb, and LVEF%. Age, sex, and BMI were also included in multiple logistical analysis because they often affect the prognosis of various diseases. ΔNTproBNP% was calculated as (NTproBNP2- NTproBNP1)/NTproBNP1*100%. A probability value (p value) less than 0.05 was considered statistically significant. All data were done using SPSS (Statistical Package for the Social Science; SPSS Inc., Chicago, IL, USA) version 22 for Microsoft Windows.

## Results

### Baseline characteristics and weaning outcome

**Fig. 1:**
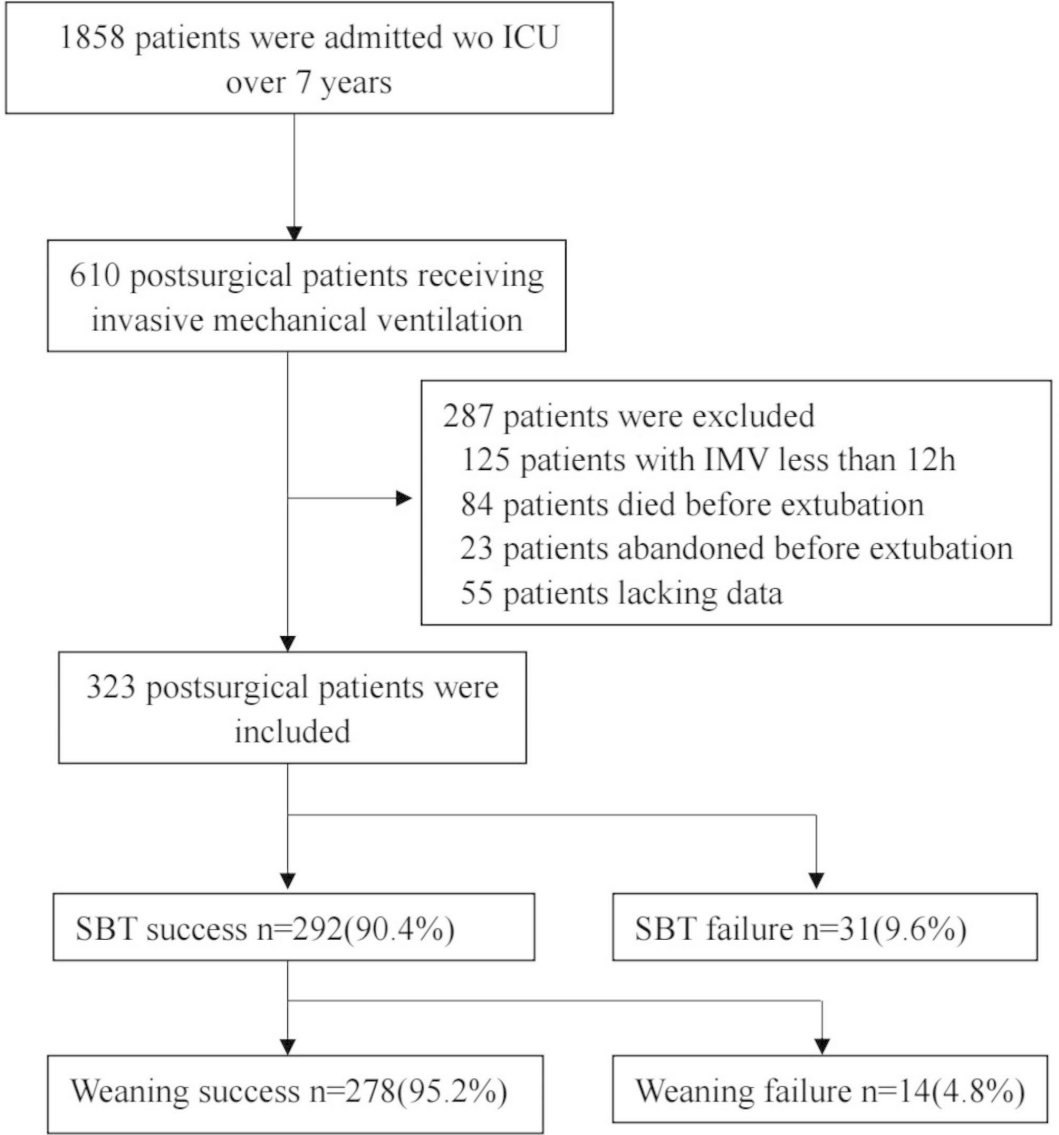
Flow chart of weaning outcomes in the study population. IMV, invasive mechanical ventilation; SBT, spontaneous breathing trial

A total of 323 postsurgical patients were included in this study, as shown in Fig. 1. Of these, 45 patients (13.9%) failed weaning (31 patients failed the SBT and 14 presented post-extubation respiratory distress and were reintubation eventually). The baseline characteristics of the study population included in this study are summarized in Table 1. Compared to weaning success group, the weaning failure group had higher APACHEII score, longer IMV duration before SBT, and longer ICU LOS. There were significant difference between the two groups in RSBI, Vte, PaO2, hemoglobin(Hb), albumin(ALB), creatinine, and β2 microglobulin, left ventricular end systolic diameter(LVDS), and left ventricular ejection fraction(LVEF%)(Table 2).

**Table 1:** Baseline characteristics

Variable	Weaning success(n = 278)	Weaning failure(n = 45)	*P*
Age(years)	71.46 ± 12.72	73.98 ± 12.71	0.218
Gender			
Male, n(%)	148(52.3)	24(53.3)	0.99
Female, n(%)	130(46.8)	21(46.7)	
BMI(kg/m^2^)	24.88(22.22–27.68)	25.06(22.05–27.58)	0.701
APACHE II score at ICU admission	16(10.75-22)	21(12–23)	0.043
Medical history, n (%)			
Chronic respiratory disorders	47(16.9)	3(6.7)	0.078
Coronary heart disease	66(23.7)	7(15.6)	0.223
Cerebral vascular disease	51(18.3)	5(11.1)	0.234
Tumor	84(30.2)	15(33.3)	0.728
Arrhythmia	18(6.5)	4(8.9)	0.526
Diabetes mellitus	92(33.1)	15(33.3)	0.975
Hypertension	162(58.3)	23(51.1)	0.368
Surgery sites, n (%)			
Intra-abdominal	185(66.5)	27(60.0)	0.391
Orthopedic	37(13.3)	7(15.6)	0.684
Urological	22(7.9)	4(8.9)	1.000
Thoracic	22(7.9)	3(6.7)	1.000
Neurologic	9(3.2)	4(8.9)	0.167
others	3(1.1)	0(0)	1.000
IMV duration before SBT (days)	1(1–2)	2(2–3)	0.002
LOS in ICU(days)	3(2-5.25)	6(4–13)	0.000
LOS in hospital(days)	23(16–31)	25(17-34.5)	0.237

Note: Continuous variables were presented as median (25th-75th percentile) or (mean ± standard deviation, SD). Categorical variables were presented as numbers

(n) and percentages (%). Difference of gender, medical history, and surgery sites between the groups were compared by Chi square (χ2) test. Difference of age was compared by Student’s t test. Difference of BMI, APACHE II score, IMV duration before SBT, LOS in ICU and LOS in hospital were compared by Mann-Whitney U-test

Abbreviation: BMI, body mass index; IMV, invasive mechanical ventilation; APACHE II, acute physiology and chronic health evaluation II; LOS, length of stay; ICU, intensive care unit; SBT, spontaneous breathing trial

### The levels of NTproBNP1, NTproBNP2 and ΔNT-proBNP%

**Fig. 2:**
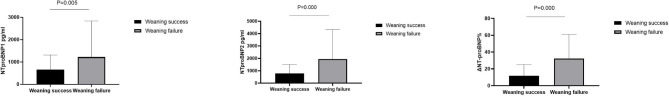
The levels of NTproBNP1, NTproBNP2 and ΔNTproBNP% in the weaning success and failure group

Compared to weaning success group, the median levels of NTproBNP1, NTproBNP2 and ΔNT-proBNP% in the weaning failure group were 1221.5pg/ml, 1946pg/ml, 32.2%, which were significantly higher than that in weaning success group (P = 0.005, P = 0.000, and P = 0.000, respectively) (Table 2; Fig. 2).

**Table 2:** Comparison of mechanical ventilation, vital signs and laboratory parameters between patients with successful and failed weaning

Variable	weaning success(n = 278)	weaning failure(n = 45)	*P*
RSBI(breaths/min/L)	36.17(31.05–42.23)	43.01(34.89–53.81)	0.001
Vte(ml)	470(425.75–488.5)	436(380.5–475.0)	0.033
RR(breaths/min)	18(16–22)	22(15.5–24.5)	0.067
MV(L/min)	7.99(6/687-8.363)	8.28(6.420–9.22)	0.256
h((beats/min)	86.76 ± 15.41	89.76 ± 12.86	0.217
SBP(mmHg)	135.10 ± 19.95	131.04 ± 18.69	0.203
SPO2	99(97–100)	98(97–100)	0.179
PH	7.44(7.404–7.470)	7.44(7.42–7.477)	0.500
PaCO2(mmHg)	36.7(32.9–41)	36(31.75–40.85)	0.432
PaO2(mmHg)	108(86–138)	100(80.6-115.5)	0.044
PaO2/FIO2	312(246.31-397.23)	276.67(231-354.76)	0.071
HB(g/L)	101(88.75-120.25)	95(84.5-108.5)	0.018
ALB(g/L)	29.54 ± 4.56	26.84 ± 4.51	0.000
Creatinine(mmol/L)	67.6(51.15–91.9)	83.8(54.55-115.75)	0.045
β2 microglobulin(mmol/L)	2.97(2.32–3.95)	3.47(2.73–6.19)	0.007
NTproBNP1(pg/ml)	651(281.65-1315.25)	1221.5(404.5-3072.5)	0.005
NTproBNP2(pg/ml)	781.5(359.13-1504.75)	1946(842.35-4334.35)	0.000
ΔNT-proBNP%	11.9(2.425–60.74)	32.2(22.286–60.74)	0.000
Long diameter of left atrium(mm)	50(46–55)	49(45–54)	0.413
Transverse diameter of left atrium(mm)	35(32–38)	35(31.5–38)	0.567
Long diameter of right atrium(mm)	46(42–49)	46(42.5–50)	0.716
Transverse diameter of right atrium(mm)	32(29–35)	32(28–36)	0.867
Transverse diameter of right ventricle(mm)	30(28–32)	30(27.5–33)	0.658
Diameter of main pulmonary artery(mm)	24(22–25)	23(22–25)	0.756
LVDS(mm)	29(27–31)	31(30–32)	0.008
LVDD(mm)	47(44–49)	47(45–49)	0.270
LVEF(%)	67(63–71)	65(62.5–68)	0.007
Fluid balance during 24 h before SBT(ml)	403(-99.38-894)	642(18.5-1285.5)	0.091

Note: Continuous variables were presented as median (25th-75th percentile) or (mean ± standard deviation, SD). Categorical variables were presented as numbers

(n) and percentages (%). Difference of gender, medical history, and surgery sites between the groups were compared by Chi square (χ2) test. Difference of HR, SBP and ALB were compared by Student’s t test. Difference of other variables were compared by Mann-Whitney U-test

Abbreviation: RSBI,rapid shallow breathing index; MV, minute volume; Vte, expired tidal volume; NT-proBNP, N-terminal prohormone B-type natriuretic peptide; HR, heart rate; RR, respiratory rate; SBP, systolic blood pressure; Hb, hemoglobin; ALB, albumin; LVDS, left ventricular end systolic diameter; LVDD, left ventricular end diastolic diameter; LVEF%, left ventricular ejection fraction;SBT, spontaneous breathing trial

### Predictive ability of NTproBNP1, NTproBNP2 and ΔNTproBNP% for weaning failure compared with other traditional weaning parameters

**Fig. 3:**
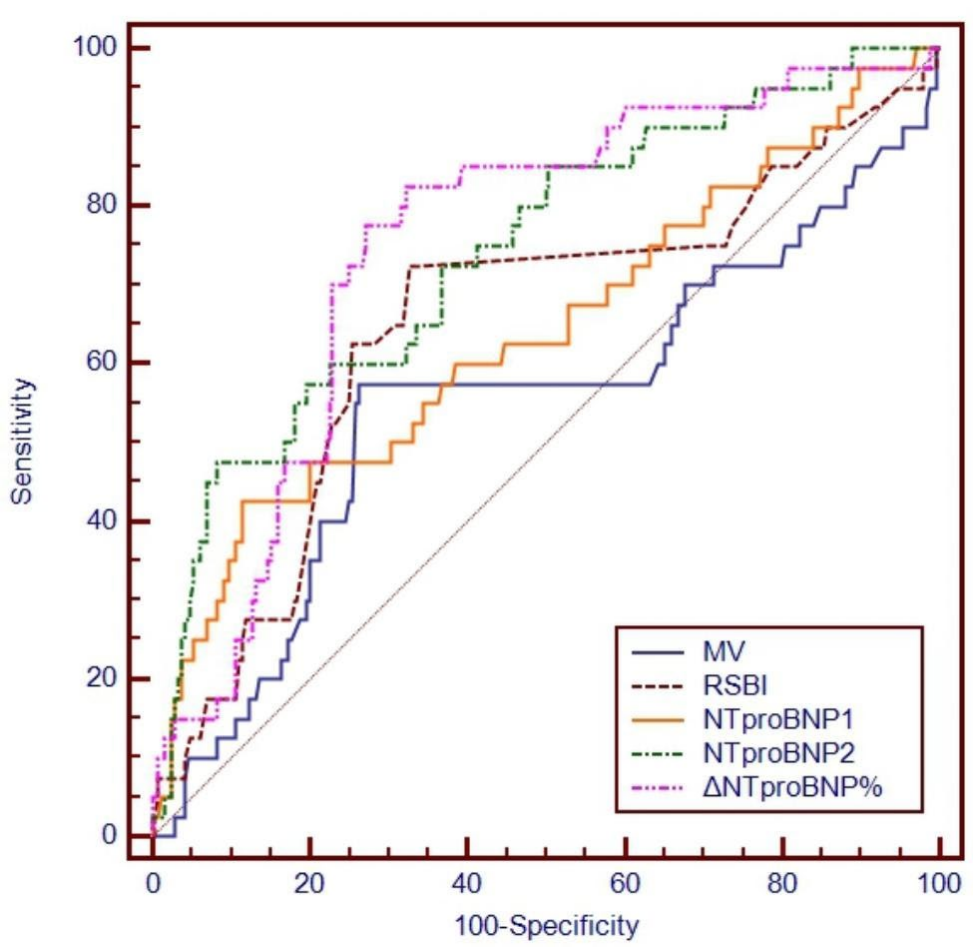
ROC curves of NTproBNP1, NTproBNP2, ΔNTproBNP%, RSBI and MV for predicting weaning failure. ROC, receiver-operating characterstic; RSBI, rapid shallow breathing index; MV, minute volume; NTproBNP, N-terminal prohormone B-type natriuretic peptide

The ROC curves of NTproBNP1, NTproBNP2 and ΔNTproBNP% and other traditional weaning parameters were shown in Fig. 3. Table 3 showed the AUC(0.744;95%CI, 0.693–0.791) of ΔNTproBNP% was higher than that of NTproBNP1(0.639;95%CI, 0.580–0.694), NTproBNP2(0.742;95%CI, 0.688–0.792), RSBI(0.651;95%CI, 0.597–0.703), and MV(0.552;95%CI, 0.496–0.607).

The cutoff value for predicting weaning failure was ΔNTproBNP%>23.3%, NTproBNP1 > 2003pg/ml, NTproBNP2 > 2610pg/ml, RSBI > 36.398 breaths/min/L, MV > 8.16 L/min. According to multiple logistical regression analysis, ΔNTproBNP%>23.3% and RSBI > 36.398 breaths/min/L were independent factors for predicting weaning failure (Table 4). Multiple logistical regression analysis was performed with covariates which showed P < 0.01 by univariate logistical analysis, including ALB, Hb, and LVEF%. Age, sex, and age were also included in the multiple logistical regression analysis because they often effect prognosis of various diseases.

**Table 3:** ROC curves of NTproBNP1, NTproBNP2, ΔNTproBNP%, RSBI and MV

Variables	NTproBNP1(pg/ml)	NTproBNP2( pg/ml)	ΔNT-proBNP%	RSBI	MV (L/min)
Cutoff value	> 2003	> 2610	> 23.3%	> 36.398	> 8.16
Sensitivity	41.46	47.5	75.56	71.11	57.78
Specificity	88.66	91.84	73.38	65.47	73.38
Likelihood ratio of positive test	3.66	5.82	2.84	2.06	2.17
Likelihood ratio of negative test	0.66	0.57	0.33	0.44	0.58
Positive predictive value,%	37.8	48.7	31.5	25	26
Negative predictive value,%	90.1	91.5	94.9	93.3	91.5
Youden’s index	0.301	0.393	0.489	0.366	0.311
AUC	0.639	0.742	0.744	0.651	0.552
95%CI	0.580 to 0.694	0.688 to 0.792	0.693 to 0.791	0.597 to 0.703	0.496 to 0.607
P	0.0075	< 0.0001	< 0.0001	0.0016	0.326

Abbreviation: AUC, area under the curve; CI, confidence interval; ROC, receiver-operating characterstic; RSBI, rapid shallow breathing index; MV, minute volume; NTproBNP, N-terminal prohormone B-type natriuretic peptide

**Table 4:** Risk factors for weaning failure

	Univariate analysis			Multivariate analysis		
	Unadjusted OR	95%CI	P	Adjusted OR	95%CI	P
ΔNT-proBNP%						
≤ 23.3%	reference			reference		
> 23.3%	8.52	4.11–17.68	0.000	13.568	4.584–40.268	0.000
BNP1, pg/ml						
≤ 2003	reference			reference		
> 2003	4.478	2.265–8.853	0.000	4.239	0.653–27.534	0.13
BNP2, pg/ml						
≤ 2610	reference			reference		
> 2610	8.096	3.951–16.588	0.000	3.844	0.725–20.378	0.114
RBSI						
≤ 36.398	reference			reference		
> 36.398	4.677	2.34–9.308	0.000	3.481	1.362–8.897	0.009
MV, L/MIN						
≤ 8.16	reference			reference		
> 8.16	3.772	1.972–7.216	0.000	2.178	0.873–5.433	0.095

Note: Multiple logistical regression analysis was performed with covariates which showed P ≤ 0.01 by univariate logistical analysis, including ALB, Hb, and LVEF%. Age, sex, and BMI were also included in multiple logistical analysis

Abbreviation: RSBI, rapid shallow breathing index; MV, minute volume; Vte, expired tidal volume; NTproBNP, N-terminal prohormone B-type natriuretic peptide; Hb, hemoglobin; ALB, albumin; LVEF%, left ventricular ejection fraction; SBT, spontaneous breathing trial

### Patients’ outcome grouped by ΔNTproBNP% cutoff value

According to the cutoff value, the patients with ΔNTproBNP%>23.3% group had longer LOS in ICU. However, there was not significant difference between the groups in LOS in hospital. Besides that, the patients with ΔNTproBNP%>23.3% had longer LVDS and lower LVEF. Fluid balance during 24 h before SBT tended to be higher in patients with ΔNTproBNP%>23.3%, although it did not reach statistical difference. The weaning failure rate was significant higher in the groups of ΔNTproBNP%>23.3% compared to the group of ΔNTproBNP%≤23.3%. Hospital mortality rate seemed to be higher in patients with ΔNTproBNP%>23.3%, although it did not reach statistical difference between the two groups (Table 5).

**Table 5:** Patients’ characteristics and outcome grouped by ΔNTproBNP% cutoff value

Variables	ΔNT-proBNP%>23.3%	ΔNT-proBNP%≤23.3%	P
	n = 108	n = 215	
LVDS(mm)	30(27–32)	29(27–31)	0.021
LVEF (%)	65(62-69.75)	67(63–71)	0.015
Fluid balance during 24 h before SBT(ml)	481(100–1047)	366(-142-931)	0.194
LOS in ICU(days)	4(3–7)	3(2–6)	0.000
LOS in hospital(days)	24.5(16.25-34)	23(16–30)	0.295
weaning failure,n(%)	34(31.485)	11(5.115)	0.000
Hospital mortality,n(%)	9(8.3)	11(5.1)	0.258

Note: Continuous variables were presented as median (25th-75th percentile), and compared by Mann-Whitney U-test

Abbreviation: LVDS, left ventricular end systolic diameter; LVEF%, left ventricular ejection fraction; SBT, spontaneous breathing trial; LOS, length of stay

## Discussion

In this study, we found that ΔNTproBNP%>23.3% with the highest AUC of ROC (0.744;95%CI, 0.693–0.791) was a more useful marker for predicting weaning failure when compared to traditional weaning parameters among postsurgical patients, and ΔNTproBNP%>23.3% was an independent risk factor for weaning failure. The adjusted OR of ΔNTproBNP%>23.3% was 13.568, indicating that the risk of weaning failure of patients with ΔNTproBNP%>23.3% was much higher than patients with ΔNTproBNP%≤23.3%.

A successful weaning from mechanical ventilation depends on adequate respiratory strength and endurance, stable hemodynamics, electrolyte balance, restored lung function but also on optimal performance of other organ systems including powerful heart function [18]. During weaning process, cardiovascular function was compromised by increases in cardiac preload and afterload caused by intrathoracic pressure shifts from positive to negative, and increases in catecholamine secretion and work of breathing [9]. Especially for patients experiencing major surgery, they tended to have insufficient cardiac function because surgeries, anaesthesia and fluid load during surgery could have some adverse effect on the cardiac function [11–13].

This leads to possible decompensated heart failure or pulmonary oedema [25]. BNP or NTproBNP has been considered as a sensitive marker of cardiovascular dysfunction and could predict weaning failure due to cardiac reason [22]. In this study, the precent change of NTproBNP was a better predictor for weaning failure than NTproBNP1 and NTproBNP2, indicating that patients with weaning failure in the current study had much more increases in NTproBNP level during 2 h SBT than patients who weaned successfully. Besides that, patients with ΔNTproBNP%>23.3% had higher LVDS and lower LVEF% compared to patients with ΔNTproBNP%≤23.3%. This suggested the postsurgical patients with ΔNTproBNP%>23.3% had reduced cardiac function. Inadequate cardiac reserve might contribute to subsequent respiratory insufficiency and weaning failure.

Some studies showed similar results. Grasso showed that an elevated NTproBNP during SBT predicted weaning-induced cardiac dysfunction among COPD patients [26]. Farghaly found that the change in plasma BNP level of < 14.9% from the pre-SBT baseline may be a good predictor of weaning success among patients with respiratory illness [18].An elevated BNP level is also considered to be a biomarker of ventricular dysfunction and can identify early decompensated heart failure after cardiac surgery patients [19].

There were some different results. Mekontso-Dessap had reported that the higher level of BNP level at baseline were associated with weaning failure but the change of BNP level during 1 h SBT could not differentiate between patients of extubation success and failure. This discrepancy might be attributed to the sampling interval of NTproBNP. Besides that, our study population focused on the patients after surgery, while Mekontso-Dessap et al. studied the medical patients.

RSBI was also an independent predictor of weaning failure in this study, however, by compared the AUC between ΔNTproBNP% and RSBI, we concluded that RSBI was an inferior predictive marker of weaning failure. Although Fadaii reported although RSBI < 105 was a helpful index for weaning success, application of RSBI alone may mislead the physicians [27]. Recent study reported that RSBI measured early during an SBT cannot accurately predict the successful outcome of a T-piece trial in a homogenous population of patients with COPD [28]. These findings could be explained by the fact that RSBI can be significantly affected by the level of ventilator support [29].Hence, RSBI may not be a good predictor of weaning outcome.

MV had been reported as a classic index to predict a successful weaning outcome [30]. Nevertheless, we found ΔNTproBNP% outmatched MV in predicting weaning failure. In line with previous study, MV could not predict weaning outcome [18].

## Limitations

There are several limitations in the present study. First, the retrospectively study in a single center with a small sample size limits the generalizability of the findings of this work, since the results may heavily depend on the type of patients and the ventilator practices. Second, there are so many factors affecting the levels of NTproBNP such as diastolic dysfunction [31], right ventricular dysfunction [32], pulmonary hypertension [32], and myocardial ischemia [33], which were not systematically assessed in our study. Third, due to lack of data, we did not evaluate the correlation of NTproBNP with other more classic parameters related with weaning, such as P 0.1, negative inspiratory force, and cough peak flow; Fourth, the AUC of 0.744 of ΔNTproBNP%>23.3% by ROC curve suggested that ΔNTproBNP% only have moderate predicting ability, and so ΔNTproBNP% should be considered together with other traditional weaning parameters to optimize weaning outcome.

## Conclusion

The present study suggested that ΔNTproBNP% during 2 h SBT is a valuable marker for predicting weaning failure than other traditional parameters among postsurgical patients, and ΔNTproBNP%>23.3% is an independent risk factor for weaning failure. The change of NTproBNP in the process of weaning can help clinicians to identify potential cardiac insufficiency in advance, and more attention should be paid on these patients during SBT. Therefore, the corresponding interventions to optimize cardiac function should be actively given to these patients, such as strengthening the monitoring of cardiac function, controlling fluid intake, improving myocardial ischemia and so on during the perioperative periods.

## Data Availability

All data generated or analyzed during this study are included in this published article.

## Abbreviations

NTproBNP: N-terminal prohormone BNP
SBT: Spontaneous breathing trial
IMV: Invasive mechanical ventilation
ICU: Intensive care unit
PaO_2_/FiO_2_: Arterial oxygen tension/fraction of inspired oxygen
RR: Respiratory rate
HR: Heart rate
SBP: Systolic blood pressure
PaCO_2_: Arterial carbon dioxide tension
SpO_2_: Peripheral oxygen saturation
NIV: Noninvasive ventilation
ROC: Receiver operator characteristic
AUC: Area under the curve
CI: Confidence interval
BMI: body mass index
APACHE II: acute physiology and chronic health evaluation II
LOS: length of stay
ICU: intensive care unit
SBT: spontaneous breathing trial
RSBI: rapid shallow breathing index
MV: minute volume
Vte: expired tidal volume
Hb: hemoglobin
ALB: albumin
LVDS: left ventricular end systolic diameter
LVDD: left ventricular end diastolic diameter
LVEF%: left ventricular ejection fraction
